# Exploring Antioxidant, Antimicrobial and Anti-Inflammatory Effects of *Juglans regia* and *Pfaffia paniculata* Extracts: Implications for Intestinal Dysbiosis and Colorectal Cancer Risk Associated with Oral Pathogens

**DOI:** 10.3390/pharmaceutics17060693

**Published:** 2025-05-25

**Authors:** Diego Garcia Miranda, Lucas de Paula Ramos, Nina Attik, Nicole Van Der Heijde Fernandes Silva, Pyetra Claro Camargo, Gabriela Ferraz de Araujo, Nicole Fernanda dos Santos Lopes, Maria Cristina Marcucci, Cristina Pacheco-Soares, Bruno Henrique Godoi, Giovanna Arruda Caires, Hugo Vigerelli, Florence Carrouel

**Affiliations:** 1 Laboratory Health Systemic Process-P2S, UR4129, Faculty of Medicine Laennec, University Claude Bernard Lyon 1, University of Lyon, 11 rue Guillaume Paradin, F-69008 Lyon, France; florence.carrouel@univ-lyon1.fr; 2 Department of Biosciences and Oral Diagnosis, Institute of Science and Technology, São Paulo State University, Francisco José Longo 777, São José dos Campos 12245-000, SP, Brazil; nicoleheijde30@gmail.com (N.V.D.H.F.S.); pyetraclaro@hotmail.com (P.C.C.); gabrielaferraz.dearaujo@gmail.com (G.F.d.A.); nf.lopes@unesp.br (N.F.d.S.L.); cristina.marcucci@unesp.br (M.C.M.); 3 Multimaterials and Interfaces Laboratory, CNRS UMR 5615, University Claude Bernard Lyon 1, University of Lyon, F-69008 Lyon, France; nina.attik@univ-lyon1.fr; 4 Anhembi Morumbi University, Avenue Deputado Benedito Matarazzo 6070, São José dos Campos 12230-002, SP, Brazil; 5 School of Dentistry, Federal University of Alfenas-UNIFAL, R. Gabriel Monteiro da Silva, 700-Centro, Alfenas 37130-001, MG, Brazil; 6 Laboratory of Cell Compartment Dynamics, Institute of Research and Development, University of Vale do Paraíba, Av. Shishima Hifumi 2911, São José dos Campos 12244-000, SP, Brazil; cpsoares@univap.br (C.P.-S.); bhenriquegodoi@gmail.com (B.H.G.); 7 Laboratory of Genetics, Butantan Institute, São Paulo 05503-900, SP, Brazil; giovanna.caires.esib@esib.butantan.gov.br; 8 Laboratório de Bioquímica, Instituto Butantan, São Paulo 05503-900, SP, Brazil; hugo.barros@butantan.gov.br

**Keywords:** colorectal neoplasms, dysbiosis, anaerobic bacteria, walnut, brazilian ginseng, antioxidant, antimicrobial agents, herbal medicine, phenolic compounds, plant extract

## Abstract

**Background/Objectives:** Colorectal neoplasms rank as the third most prevalent cancer globally and stand as the second leading cause of cancer-related mortality. Its etiology is multifaceted, pointing to the role of microorganisms within the human microbiota in its development. Notably, the high prevalence of oral pathogens like *Fusobacterium nucleatum* and *Parvimonas micra* is implicated in inducing gut dysbiosis and stimulating the proliferation and metastasis of cancer cells. Therefore, this study aimed to evaluate in vitro the biological effects of extracts from *Juglans regia* and *Pfaffia paniculata*. **Methods:** Phytochemical analysis was carried out by HPLC, and the antioxidant effect was determined by DPPH. Antimicrobial activity was investigated on *F. nucleatum* and *P. micra* planktonic and biofilms. Metabolic activity and genotoxicity were performed. **Results:** *J. regia* and *P. paniculata* expressed CE50 37.26 and 1367.57 mcg, respectively. The extracts exhibited a minimum bactericidal concentration of 1.73 and 0.48 mg/mL for *J. regia* and *P. paniculata*, respectively. Reduction superiorly 90% of *P. micra* biofilms. Metabolic activity was varied proportionally to the extract concentration, and no genotoxic effects were observed. **Conclusions:** The *J. regia* extract has great antioxidant activity and could be used as an alternative in combating pathogens associated with the onset of dysbiosis and tumor progression in colorectal neoplasms. Nevertheless, further studies are needed to validate their clinical applicability.

## 1. Introduction

Worldwide, 1 in 6 deaths is due to cancer [1]. Colorectal neoplasms are the third most incident cancer in the world and the second in terms of mortality, with around 1.8 million new cases and more than 800,000 deaths in 2018 [2] and 1 million in 2020 [3].

The etiology of colorectal cancer is complex since the development of this cancer is due to the interaction of various factors, both genetic and environmental [4]. Among the genetic factors, hereditary diseases most associated with cancer development include Lynch syndrome and familial adenomatous polyposis [5]. In relation to environmental factors, according to the World Cancer Research Fund/American Institute for Cancer Research, they are the consumption of processed meats, alcohol, and obesity [2]. There is also scientific evidence of the influence of microorganisms from the human microbiota on the development of colorectal cancer [5,6].

The human microbiota comprises various microorganisms, including bacteria, viruses, fungi, protozoa, and helminths [7]. These microorganisms are important for ensuring the homeostatic control of the body. However, the loss of this balance, also known as dysbiosis, can result in inflammation, intestinal barrier failure, damage to mucosal tissue, and positive upper regulation of oncogenes. These mechanisms are implicated in the development of various diseases, including colorectal cancer [7].

Recent studies have shown differences in the intestinal microbiota of healthy patients compared to patients with colorectal cancer [1,8,9]. Therefore, the abundant presence of some microorganisms, especially bacteria, causes intestinal dysbiosis and stimulates the growth and metastasis of cancer cells [10].

Among the bacteria strongly associated with the development of colorectal cancer are *Fusobacterium nucleatum* [11] and *Parvimonas micra* [12]. Both are anaerobic bacteria and are commonly found in the oral cavity [13,14]. Their presence in the oral cavity is associated with intestinal dysbiosis [15,16]. This could be due to bacteremia caused by dental procedures like tooth brushing, flossing, tooth extraction, teeth cleaning, and periodontal surgeries [7,17,18]. Importantly, *F. nucleatum* and *P. micra* are frequently detected in colorectal cancer tissues. Quantitatively, *F. nucleatum* has been reported in up to 40–45% of colorectal cancer tumors, with notably higher abundance in the proximal colon. P. micra has been detected in 27–35% of tumor samples, with one study reporting an increase from 8.7% in adenomas to 25.4% in carcinomas—highlighting its potential involvement in late-stage tumorigenesis. These prevalence data reinforce their significance not only as microbial biomarkers but also as potential therapeutic targets for colorectal cancer prevention and management [19,20,21,22].

A way to fight against these bacteria that are risk factors for cancer could be antibiotics. Indeed, recent animal studies have demonstrated that administering antibiotics to mice infected with such bacteria reduced tumor growth, suggesting a potential therapeutic approach to controlling these pathogenic agents [1]. However, antimicrobial resistance presents a significant challenge in eradicating bacterial infections that are precursors to cancer and are a major contributor to therapeutic failures [23]. Therefore, there is a critical need to develop novel therapies, such as phytotherapy to control bacterial proliferation to reduce dysbiosis and the risk of colorectal cancer development.

Among the components of phytotherapy, *Juglans regia*, known as common walnut, has some therapeutic properties such as anti-inflammatory [24], antioxidant [25], and antimicrobial activity [26]. Farooqui et al. [27] demonstrated the spectrum of action of its methanolic extract on enteric *Salmonella Typhi*, *Salmonella Paratyphi* A, *Acinetobacter baumannii*, *Klebsiella pneumoniae*, *Pseudomonas aeruginosa*, *Helicobacter pylori*, *Shigella species*, *Campylobacter jejuni*, and *Escherichia coli*, showing a minimum inhibitory concentration against multi-resistant clinical strains. *J. regia* is also credited with antineoplastic activity, as demonstrated in the study by Li et al. [28], in which the ethanolic extract promotes a decrease in the growth rate of human esophageal neoplastic cells, using the KYSE150 and EC9706 strains. The researchers point out that several proteins known as markers of neoplastic alteration are reduced after the application of the extract, as well as promoting cell apoptosis.

Furthermore, *Pfaffia paniculata*, popularly known as Brazilian Ginseng, is a root popularly used for the treatment of various diseases such as sickle cell disease [29], gastric disorders [30,31], diabetes, rheumatism, and especially as an invigorating tonic [32]. Scientific research only focuses on a few branches of action of the extract, such as the anti-inflammatory potential and the ability to fight tumor cells. Da Silva et al. [32] evaluated the action of the root extract on rats with hepatocarcinoma. The animals treated with the *P. paniculata* extract showed a decrease in tumor cell proliferation and an increase in tumor cell apoptosis. Nagamine et al. [33], who analyzed the action of the butanolic extract on the MCF-7 neoplastic cell line, found severe morphological deformations in the cells, with alterations in the cytoplasmic and nuclear components. Costa et al. [31] evaluated the anti-inflammatory effect of the *P. paniculata* extract by inducing an inflammatory bowel disease in rats, which were then treated for 14 days with doses of the root extract, with the treatment showing a reduction in the levels of the cytokines IL-1β, INF-γ, TNF-α, and IL-6.

In this context, the current study aims to assess the biological effects of *J. regia* and *P. paniculata* glycolic extracts to propose a future new method in the treatment of dysbiosis correlated with colorectal cancer. To this end, the antioxidant, metabolic activity, genotoxicity, and antimicrobial actions of *F. nucleatum* and *P. micra* were analyzed in vitro.

## 2. Materials and Methods

### 2.1. Chemical Reagents

Glycolic extract of *Juglans regia* (CAS n°: 84012-43-1; lot: PRODO18746, Mapric Greentech company^®^, São Paulo, Brazil); glycolic extract of *Pfaffia paniculata* (lot: PRODO19544, Mapric Greentech company^®^); aluminum chloride (CAS n°: 7446-70-0, 98% purity, Sigma-Aldrich^®^, St. Louis, MO, USA); ethanol (CAS n°: 64-17-5, 99.5% purity,: Synth^®^, Diadema, Brazil); Folin–Ciocalteau reagent (Sigma-Aldrich^®^, St. Louis, MO, USA); sodium carbonate (CAS n°: 497-19-8, 99% purity, Sigma-Aldrich^®^); methanol (CAS n°: 67-56-1, purity: 99.8% Synth^®^); formic acid (CAS n°: 64-18-6, 98% purity, Sigma-Aldrich^®^); diphenyl picrylhydrazyl radical (DPPH) (CAS n°: 1898-66-4, 100% purity, Sigma-Aldrich^®^); brucella broth and agar (Becton Dickinson^®^, Franklin Lakes, NJ, USA); hemin (CAS n°: 16009-13-5, 96% purity, Sigma-Aldrich^®^); vitamin K (CAS n°: 58-27-5, 99.8% purity, Sigma-Aldrich^®^); fetal bovine serum (FBS) (Invitrogen^®^, New York, NY, USA); sterile saline solution (0.9% NaCl) (LGC Biotechnology^®^, Cotia, Brazil); 3-(4,5-Dimethyl-2-thiazolyl)-2,5-diphenyl-2H-tetrazolium bromide powder (MTT) (CAS n°: 298-93-1, 97.5% purity, Sigma-Aldrich^®^); Eagle’s medium modified by Dulbecco (DMEM) (LGC Biotechnology^®^); dimethyl sulfoxide (DMSO) (CAS n°: 67-68-5, 99.9% purity, Sigma-Aldrich^®^); ethyl methane sulfonate (EMS) (CAS n°: 62-50-0, Sigma-Aldrich^®^); cytochalasin B (CAS n°: 14930-96-2, purity: 98%, Sigma-Aldrich^®^); phosphate-buffer saline (PBS) (Sigma-Aldrich^®^); DAPI fluoroshield (CAS n°: 28718-90-3, Sigma-Aldrich^®^).

### 2.2. Equipment

Analytical balance (Balance XPR106DUH/A, Mettler Toledo^®^, Columbus, OH, USA); Water bath precision (TSGP02, Termo Fisher Scientific^®^, Waltham, MA, USA); drying and sterilization oven (CQA Química Americana LTDA^®^, Paulinia, São Paulo, Brazil); stirrer (Micro plate shaker MIX-1500, Miulab^®^, Hangzhou, China); spectrophotometer (ELX808LBS, Lonza Biotek^®^, Winooski, VT, USA); high-performance liquid chromatography with a photodiode detector instrument—HPLC DAD (Merck-Hitachi D-7000^®^, Tokyo, Japan); LiChrospher^®^ RP-18 HPLC column, 5 µm particle size, L × I.D. 12.5 cm × 4.6 mm from (Merck, Darmstadt, Germany); anaerobic chamber (Don Whitley Scientific Limited^®^, Whitley DG250 Workstation, Shipley, West Yorkshire, UK); ultrasonic homogenizer (Biosystems^®^, LUHS-A10-1C, Curitiba, Parana, Brazil); CO_2_ incubator (MCO-19AIC (UV, Sanyo^®^) Osaka, Japan); fluorescence microscope (DFC310FX, Leica Microsystems^®^, Wetzlar, Hessen, Germany).

### 2.3. Soluble Solids Content in Ethanol

Three 25 mL beakers were weighed on an analytical balance, and the weights were noted. 5 mL of the extract was pipetted into each beaker and left to dry in a drying and sterilization oven at 80 °C. Once dry, it was placed in a desiccator until it cooled and then weighed. The amount of soluble solids in the extract was calculated:

% sol. soluble (*w/w*) = % sol. soluble (*w/v*)/density

### 2.4. Determination of Total Phenol Content

To prepare the stock solution, 1 mL of each extract was transferred to a 100 mL volumetric flask, mixed with 4 mL of ethanol, and brought to volume with 95 mL of distilled water under constant stirring. All subsequent steps were conducted in triplicate. In a separate 10 mL volumetric flask, 5 mL of distilled water, 800 μL of Folin–Ciocalteu reagent, and 200 μL of the stock solution were combined. The mixture was stirred, followed by the addition of 1.2 mL of a 20% sodium carbonate solution. The flask was then filled to the mark with distilled water. The resulting solution was incubated in a water bath at 20 °C. After a 2 h reaction period, the final volume was confirmed at 20 °C, the solution was mixed again, and absorbance was measured at 760 nm using a spectrophotometer. The value of total phenols was determined by linear regression using gallic acid as a standard (calibration curve).

### 2.5. Determination of the Total Flavonoid Content Expressed as Quercetin in Juglans regia and Pfaffia paniculata Extracts

To quantify the total flavonoid content in the extracts, a stock solution was prepared by adding 100 μL of the glycolic extract to a 10 mL volumetric flask and filling it to the mark with methanol. All subsequent steps were carried out in triplicate. From the stock solution, a 200 μL aliquot was taken and transferred to another 10 mL flask already containing 5 mL of methanol. Then, 200 μL of aluminum chloride (AlCl_3_) solution was added, and the volume was adjusted to approximately 10 mL with methanol. The mixture was stirred and incubated in a water bath at 20 °C for 30 min. After incubation, the final volume was adjusted, and absorbance was measured at 425 nm. The total flavonoid content was calculated by linear regression using a quercetin calibration curve and expressed as quercetin equivalents.

### 2.6. Phytochemical Analysis of Juglans regia and Pfaffia paniculata Extracts by High-Performance Liquid Chromatography with Diode-Array Detection

HPLC-DAD was used to characterize the marker content profile in the extracts. The chromatographic conditions were the mobile phase composed of water-formic acid solution diluted in a ratio of 95:5 (solvent A) and chromatographic-Merck grade methanol (solvent B), and the stationary phase was a LiChrospher RP-18 HPLC column, 5 µm particle size, L × I.D. 12.5 cm × 4.6 mm. The flow was 1 mL/min and a linear gradient starting with 0% of solvent B, ending with 70% of solvent B, in a run time of 50 min. The detection wavelengths used were 280 and 340 nm.

### 2.7. Evaluation of Antioxidant Activity of Juglans regia and Pfaffia paniculata Extracts

A total of eleven tubes, labeled 0 through 10, were prepared. Each tube received 1 mL of a 0.30 mM DPPH solution in ethanol and 1 mL of the wild-type extract diluted to specific concentrations in ethanol as follows: tube 1: 0.01%, tube 2: 0.005%, tube 3: 0.0025%, tube 4: 0.00125%, tube 5: 0.000625%, tube 6: 0.0003125%, tube 7: 0.00015625%, tube 8: 0.00007812%, tube 9: 0.00003906%, and tube 10: 0.00001953%. Tube 0, which contained only the DPPH solution, served as the blank for spectrophotometer calibration. After mixing for 1 min, the tubes were left to react, and absorbance was recorded at 515 nm after 30 min. A graph was plotted with the percentage of DPPH inhibition (A%) on the *y*-axis against extract concentration (µg/mL) on the *x*-axis. The EC_50_ value (µg/mL) was determined using the least-squares regression method in a spreadsheet program.

### 2.8. Determination of the Minimum Inhibitory Concentration (MIC) and Minimum Bactericidal Concentration (MBC) of Juglans regia and Pfaffia paniculata Extracts by CLSI M11-A7

Strains of *F. nucleatum* (ATCC 25586) and *P. micra* (ATCC 33270) were cultured in enriched in Brucella agar containing 1% hemin, and 1% vitamin K at 37 °C for 48 h in an anaerobic chamber. For each strain, bacterial inoculums were prepared in sterilized saline solution and standardized at 1 × 10^8^ colony forming unit (CFU)/mL according to the MacFarland scale.

In parallel, serial dilutions of *J. regia* (initial concentrations: 1.73 mg/mL) and *P. paniculata* (initial concentrations: 0.48 mg/mL) extracts were prepared in microplates. A total of 10 successive 1:2 dilutions were performed using 100 µL Brucella broth medium.

Later, 100 µL of standardized inoculum was added to each well. After incubation for 48 h at 37 °C, MIC values were determined. It corresponded to the concentration in the first well, with the absence of microbial turbidity, next to the well with apparent microbial growth.

Finally, MBC values were determined by inoculating a 10 µL aliquot of each well into Brucella agar. After incubation for 48 h at 37 °C, MBC corresponded to the well with no grown bacteria and with the lowest concentration of *J. regia* and *P. paniculata* extracts.

### 2.9. Antibiofilm Action of Juglans regia and Pfaffia paniculata Extracts

Biofilms were established with bacterial inoculum concentration at 1 × 10^8^ CFU/mL for seven days. Following biofilm formation, the supernatant was discarded, and the biofilms were treated with *J. regia* 6.92, 3.46, and 1.73 mg/mL and *P. paniculata* extract at 1.93, 0.96, and 0.48 mg/mL for a duration of 5 min. Additionally, treatments carried out for 24 h were applied at concentrations of 3.46, 1.73, and 0.86 mg/mL for *J. regia* and concentrations of 0.96, 0.48, and 0.24 mg/mL for *P. paniculata*. A total of 10 replicates were performed per experimental group. To remove the affected bacterial cells, the wells were washed with a sterilized saline solution. The biofilms were disaggregated with an ultrasonic homogenizer operating at a power of 25%. Aliquots were drawn from the microplates for dilutions of 10^−2^, 10^−4^, and 10^−6^ before being seeded on Brucella agar at a volume of 10 μL, followed by incubation in an anaerobic chamber for 48 h. After the incubation period, the plates were submitted to CFU counting.

### 2.10. Metabolic Activity Assessment of Juglans regia and Pfaffia paniculata Extracts on Human Keratinocytes (HaCaT)

The metabolic activity analysis of *J. regia* and *P. paniculata* extracts was carried out on HaCaT cells cultured in DMEM, with a high concentration of glucose (4.5 g/L), supplemented with 10% FBS, and incubated at 37 °C, atmospheric humidity, and 5% CO_2_ exposed to the extracts for 5 min and 24 h.

The metabolic activity was assessed using the MTT colorimetric assay, which relies on enzymatic reduction by metabolically active cells. A total of 2 × 10^4^ viable cells per well were seeded into 96-well plates containing 200 µL of DMEM supplemented with 10% fetal bovine serum (FBS), and incubated at 37 °C in a 5% CO_2_ atmosphere for 24 h to promote cell attachment. After this period, cells were treated with five different concentrations of the extracts for either 5 min or 24 h. DMEM with 10% FBS served as the negative control. Following treatment, the MTT assay was carried out by adding 100 µL per well of a 0.5 mg/mL MTT solution prepared in DMEM with 10% FBS. The plates were then incubated in the dark for 4 h under standard conditions (37 °C, 5% CO_2_). The medium was removed, and 100 µL of DMSO was added to each well to solubilize the formazan crystals. After 10 min of incubation with agitation, absorbance was measured at 570 nm using a spectrophotometer. Cell viability was expressed as a percentage relative to the untreated control, considered as 100%.

### 2.11. Micronucleus Test of J. regia and P. paniculata Extracts on Human Keratinocytes (HaCaT)

HaCaT at a concentration of 3 × 10^5^ cells/mL was cultured in 96-well microplates with 1 mL of DMEM supplemented with 10% SFB for 24 h at 37 °C in a 5% CO_2_ atmosphere. The cells were exposed to the experimental groups, with the extracts diluted in DMEM supplemented with 10% SFB at concentrations of 0.108 and 0.054 mg/mL for *J. regia* and 0.48 and 0.24 mg/mL for *P. paniculata*. The negative control group received only the culture medium, while the positive control group received EMS at a concentration of 5 mM; both treatments were applied for 24 h.

Following treatment, cells were rinsed three times with PBS and then incubated with cytochalasin B (6 μg/mL) for 24 h at 37 °C in an atmosphere containing 5% CO_2_. After incubation, cells were fixed using 100% methanol for 20 min and subsequently stained with DAPI. The staining solution was removed after 5 min of exposure, and cells were washed three times with PBS. Micronuclei were observed using a fluorescence microscope at 40× magnification, with a total of 2000 cells analyzed per well.

### 2.12. Statistical Analysis

The data obtained was analyzed for normality using the D’Agostino, Shapiro–Wilk, and Kolmogorov–Smirnov tests. For data exhibiting a normal distribution, one-way ANOVA followed by Tukey’s post hoc test was applied. Non-normally distributed data were evaluated using the Kruskal–Wallis test, followed by Dunn’s multiple comparisons test. Statistical significance was defined as follows: *p* < 0.0332 (*), *p* < 0.0021 (**), *p* < 0.0002 (***), *p* < 0.0001 (****). All statistical analyses were performed using GraphPad Prism 9.0 software.

## 3. Results

### 3.1. Physicochemical and HPLC-DAD Analysis of J. regia and P. paniculata Extracts

The results of the phytochemical analysis of *J. regia* and *P. paniculata* extracts are shown in Table 1. *J. regia* showed a higher concentration of phenols and a lower concentration of flavonoids when compared to *P. paniculata.*

**Figure 1:**
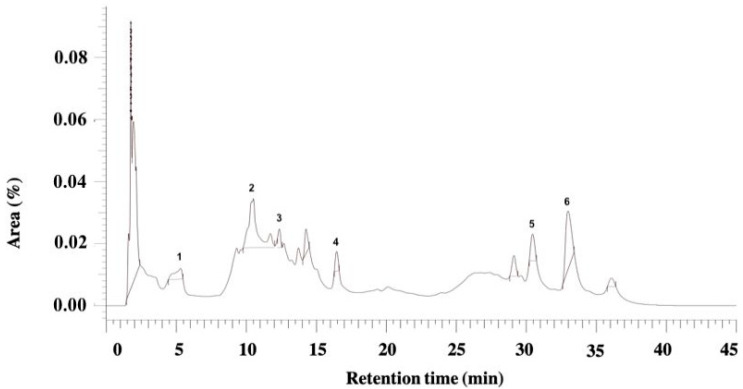
HPLC-DAD chromatogram of the glycolic extract of *J. regia.* **1**: caffeic acid derivative (Rt = 5.31 min); **2**: quercetin derivative (Rt = 10.50 min); **3**: caffeic acid derivative (Rt = 12.35 min); **4**: *O*-heteroside of quercetin (Rt = 16.46 min); **5**: p-coumaric acid derivative (Rt = 30.45 min); **6**: caffeoylquinic acid (Rt = 32.97 min).

In HPLC-DAD analyses, *J. regia* extract showed the presence of caffeic acid derivative (Retention time (Rt) = 5.31 min), quercetin derivative (Rt = 10.50 min), caffeic acid derivative (Rt = 12.35 min), O-heteroside of quercetin (Rt = 16.46 min), p-coumaric acid derivative (Rt = 30.45 min), and caffeoylquinic acid (Rt = 32.97 min) [34] (Figure 1).

**Figure 2:**
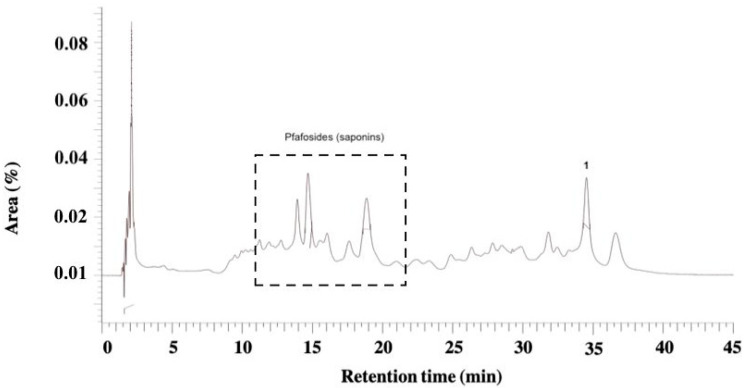
HPLC chromatogram of the glycolic extract of *P. paniculata.* A group of saponins (between 10 and 20 min of Rt) [35] and pfaffic acid Rt 34.53 min [36].

HPLC-DAD analyses of *P. paniculata* extract showed the presence of benzophenone derivatives at Rt of 9.00, 17.40, and 18.86 min. The glycoside, pfaffic acid, was present (Rt = 14.69 min) (Figure 2).

### 3.2. Antioxidant Activity of J. regia and P. paniculata Extracts

Table 2 analyzes the antioxidant activity of *J. regia* and *P. paniculata* extracts. *J. regia* exhibited a greater antioxidant effect than *P. paniculata*.

### 3.3. Minimum Inhibitory Concentration and Minimum Bactericidal Concentration of J. regia and P. paniculata Extracts

The MBC exhibited the same value for both *F. nucleatum* and *P. micra* bacteria (Table 3). The MIC was not determined due to the turbidity of the broth.

### 3.4. Evaluation of Antibiofilm Action of J. regia and P. paniculata Extracts

The extract of *J. regia* significantly reduced bacterial biofilms in a concentration- and time-dependent manner. After 5 min of exposure, it decreased *F. nucleatum* biofilm by 14.9%, 64.1%, and 83.8% at concentrations of 1.73, 3.46, and 6.92 mg/mL, respectively. A 24 h treatment led to reductions of 13.4%, 43.0%, and 83.7% at lower concentrations (0.86, 1.73, and 3.46 mg/mL). Against *P. micra, J. regia* was highly effective, achieving over 98.3% biofilm reduction at all tested concentrations (0.86–6.92 mg/mL) after just 5 min or 24 h.

**Figure 3:**
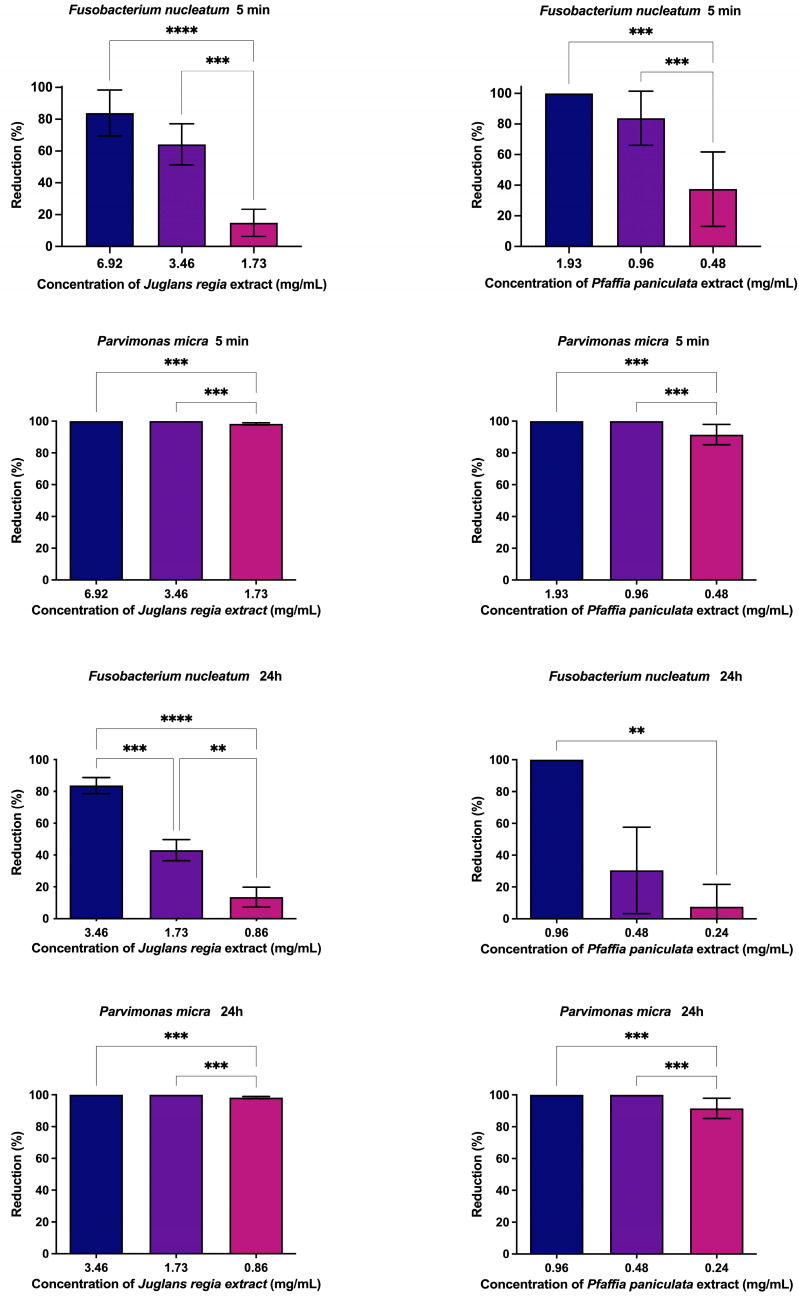
Antibiofilm action of *J. regia* and *P. paniculata* extracts at 5 min and 24 h, *p* < 0.0021 (**), *p* < 0.0002 (***), *p* < 0.0001 (****).

Similarly, *P. paniculata* extract demonstrated strong antibiofilm activity. A 5 min application reduced *F. nucleatum* biofilm by 37.5% (0.48 mg/mL), 83.95% (0.96 mg/mL), and 100% (1.93 mg/mL), while 24 h exposure resulted in 7.52% (0.24 mg/mL), 30.40% (0.48 mg/mL), and 100% (0.96 mg/mL) reductions. Notably, *P. paniculata* also eliminated *P. micra* biofilms by more than 91.5% across all concentrations and exposure times (Figure 3).

### 3.5. Metabolic Activity Assessment of J. regia and P. paniculata Extracts by MTT Assay on Human Keratinocytes (HaCat)

**Figure 4:**
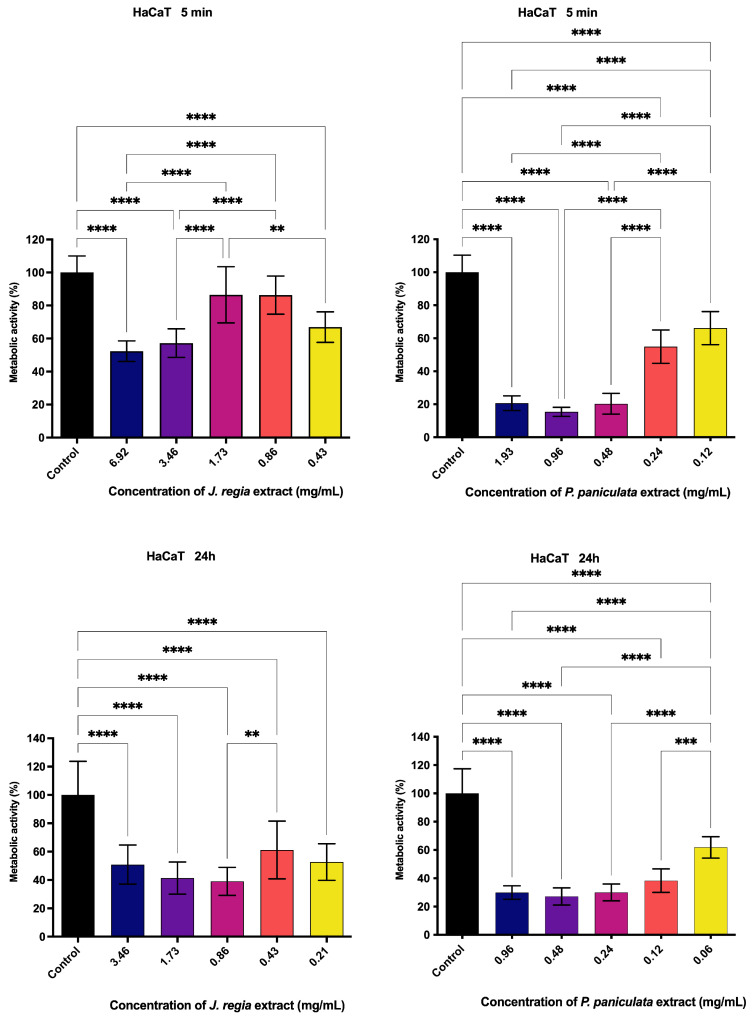
Metabolic activity of *J. regia* and *P. paniculata* on HaCat, *p* < 0.0021 (**), *p* < 0.0002 (***), *p* < 0.0001 (****).

The metabolic activity of HaCat keratinocytes treated with *J. regia* extract decreased in a dose- and time-dependent manner. After 5 min of treatment, higher concentrations of 6.92 and 3.46 mg/mL reduced metabolic activity to 52.3% and 57.2%, respectively, indicating cytotoxic effects, whereas lower concentrations (1.73, 0.86, and 0.43 mg/mL) maintained higher viability levels (86.5%, 86.3%, and 66.9%). Following 24 h of exposure, metabolic activity further declined across all concentrations, with values ranging from 50.8% to 39.0% at concentrations between 3.46 and 0.86 mg/mL, suggesting increased cytotoxicity with prolonged exposure (Figure 4).

Similarly, *P. paniculata* extract also impaired HaCat keratinocyte metabolism in a dose- and time-dependent manner. A 5 min application at concentrations of 1.93, 0.96, 0.48, 0.24, and 0.12 mg/mL led to metabolic activity levels of 20.63%, 15.28%, 20.20%, 54.8%, and 63.13%, respectively. After 24 h, concentrations of 0.96, 0.48, 0.24, 0.12, and 0.06 mg/mL resulted in metabolic activity of 29.9%, 27.16%, 29.9%, 38.3%, and 61.8% (Figure 4).

### 3.6. Genotoxicity of J. regia and P. paniculata Extracts Evaluation

**Figure 5:**
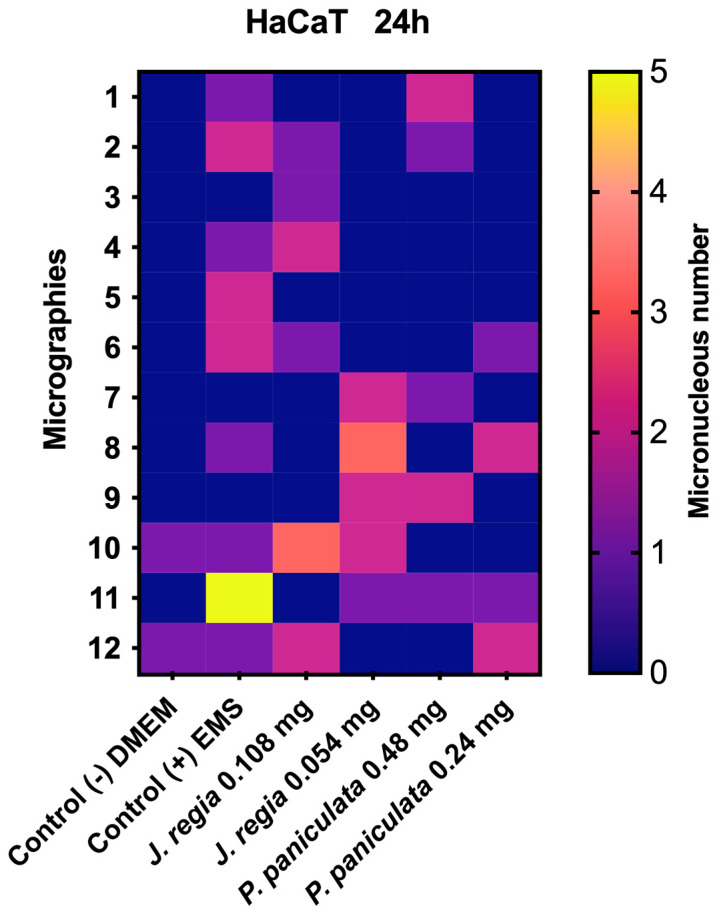
Genotoxicity assay on HaCat cells. The micrographs are represented by the heatmap rows, while the columns represent the groups tested. The number of micronuclei is shown in color, with blue (0) being the best score and yellow (5) the worst.

The application of *J. regia* extract on human keratinocytes resulted in the formation of 10 micronuclei for both concentrations tested. There was no statistical difference observed between the groups when compared to each other. The application of *P. paniculata* extract at concentrations of 0.48 and 0.24 mg/mL for 24 h on HaCat cells resulted in the formation of 11 and 8 micronuclei, respectively, in a total count of 2000 cells. Statistical analysis shows that the 0.48 and 0.24 mg/mL concentrations are statistically like the control group (Figure 5).

## 4. Discussion

The aim of this study was to evaluate the biological activity of the glycolic extracts of *J. regia* and *P. paniculata* and to correlate them with the treatment of intestinal dysbiosis and a reduction in the risk of colorectal cancer. To this end, the phytochemical composition, antioxidant performance, antimicrobial action, metabolic activity, and genotoxicity of the extracts were analyzed.

The phytochemical composition showed the presence of caffeic acid, caffeoylquinic acid, p-coumaric acid, quercetin, and O-heteroside of quercetin derivatives in the *J. regia* extract. The extract of *P. paniculata* showed the presence of benzophenone, pfaffic acid, and pfafosides (saponins).

Caffeoylquinic acid, a phenolic compound, has antioxidant [37,38,39] and antimicrobial actions [40,41,42] described in the literature. Naveed et al. [41] indicated that chlorogenic acid, an isomer of caffeoylquinic acid, acts as an antimicrobial agent against species of *Klebsiella pneumoniae*, *Helicobacter pylori, Escherichia coli, Staphylococcus epidermidis, Staphylococcus aureus*, and *Stenotrophomonas maltophilia* resistant to trimethoprim/sulfamethoxazole. Fiamegos et al. [42] demonstrated inhibition of the efflux pump in wild strains of *S. aureus* and *Enterococcus faecalis*.

P-coumaric acid, in turn, has antioxidant [43], antifungal [44], antiparasitic [45], and antibacterial activity [46,47]. Benzophenone exhibits antioxidant [48], antiviral [49], antifungal [50], and antibacterial action [51,52]. Finally, saponins have antifungal [53] and antibacterial activity [54,55,56,57].

Regarding antioxidant activity, this study reported an EC_50_ of 37.26 μg/mL for *J. regia*, indicating a strong antioxidant potential comparable to standard antioxidants such as ascorbic acid. In contrast, *P. paniculata* showed a significantly higher EC_50_ of 1367.57 μg/mL, suggesting a much lower antioxidant efficacy. Bezerra et al. [58] claimed that antioxidant activity is related to the concentration of phenols present in the extract. However, in our study, *J. regia* had a lower concentration of phenols and higher antioxidant activity when compared to *P. paniculata*. Believes that this may be due to the difference in the potency of the antioxidant effects between the types of phenols. Zurek et al. [59] showed that the methanolic extract of the flower of *J. regia* produced an EC_50_ of 22.34 μg/mL, a value like that found in our study. It also showed that the *J. regia* extract had similar activity to ascorbic acid, the gold standard in antioxidant activity.

The evaluation of the antioxidant activity of *P. paniculata* extract is scarce in the literature. Eberlin et al. [60] evaluated the application of a compound formulated from *Pfaffia paniculata*, *Ptychopetalum olacoides B.*, and *Lilium candidum L*. The compound applied at concentrations of 2.5 and 5 mg/mL, on keratinocyte culture, promoted an increase of 5 U/mL of the enzyme superoxide dismutase, regardless of whether or not it was exposed to inflammatory stimulation by LPS. Also, benzophenones are responsible for improving the effects of antioxidant enzymes such as superoxide dismutase, glutathione s-transferase, and glutathione reduction [61].

The present study is the first to evaluate the antimicrobial effects of *J. regia* and *P. paniculata* extracts against the anaerobic bacteria *F. nucleatum* and *P. micra*. A MBC of 1.73 mg/mL was observed for the *J. regia* extract and one of 0.48 mg/mL for the *P. paniculata* extract, indicating that both extracts possess effective bactericidal activity at relatively low concentrations, with *P. paniculata* demonstrating greater potency. Mohammed et al. [62] verified the antibacterial action of the aqueous extract of *J. regia* at a concentration of 20 mg/mL on another anaerobic species, *Porphyromonas gingivalis.* They demonstrated using the proteomic analysis that the inhibition was due to the disruption of enzymes such as ATP synthase, NADPH dehydrogenase, and enzymes involved in fatty acid biosynthesis. Therefore, it can be imagined that the inhibition mechanisms of *F. nucleatum* and *P. micra* are like those presented for *P. gingivalis*. However, more studies are needed to determine the exact mechanism of action of the compounds present in the extract.

The mechanism that may justify the antimicrobial action of the *P. paniculata* extract on *F. nucleatum* and *P. micra* consists of the interaction between the Saponias and the plasma membrane of the bacteria. Glycosylated triterpenes (saponins) are composed of 5 sugar molecules capable of interacting with sterols present in the cell membrane of bacteria. The saponin-steroid interaction promotes rearrangements in the membrane structure, modifying its permeability [31,63,64]. These findings lead us to believe that the saponin-sterol interaction should be addressed in future studies, as this could be a key mechanism for the herbal medicine’s antimicrobial action on anaerobes, given that anaerobes need to acquire carbon elements in their metabolism, including carbohydrates, which are used for energy maintenance. The possible search for these elements in the intestinal lumen could lead to the saponin being taken up by the bacteria, destabilizing the cytoplasmic membrane [64,65,66].

Rahamouz-Haghighi et al. [67] verified the antimicrobial potential of *P. paniculata*, formerly known as *Hebanthe eriantha*, on planktonic cultures of *Staphylococcus aureus* and *Proteus vulgaris*, obtaining a MIC of 500 µg/mL. The authors also revealed the root’s antitumor activity on colon cancer cells (HCT116) with 272.6 µg/mL of the methanolic extract. These results are in line with the present study, where the antimicrobial activity was expanded by acting on anaerobic pathogens correlated with the development of colorectal cancer. It is also worth noting that the antitumor activity reported by Rahamouz-Haghighi et al. [67] corroborates the therapeutic objective of the present study, in which the combination of antimicrobial activity, combined with the reduction or elimination of pathogens, further supports the clinical investigation of this possible drug.

Regarding *P. micra*, recent studies have shown that the phylotype most associated with the development of colorectal cancer is type A. This phylotype is characterized by its hemolytic abilities and adherence properties, enabling it to colonize the gastrointestinal mucosa and induce genetic changes to the host’s DNA, thus creating a carcinogenic environment conducive to tumor development [12]. Indeed, *F. nucleatum* is commonly detected in colorectal cancer tissues, particularly in the proximal colon [3]. Thus, considering that infectious processes contribute to 16% of the causes of colorectal cancer [68].

The results obtained regarding anaerobic microorganisms are of significant clinical relevance, particularly considering the current limited therapeutic options available against infectious agents implicated in the development and progression of colorectal cancer. This scarcity is largely due to high rates of bacterial resistance and an increase in intestinal dysbiosis.

This study presents several limitations. First, the in vitro nature of the pharmacodynamic assessments limits the ability to predict the in vivo behavior of the *J. regia* and *P. paniculata* extracts. While the results offer valuable preliminary insights into their antimicrobial potential, further in vivo and clinical investigations are necessary to support their therapeutic application. Second, the evaluation of antioxidant activity was limited to the DPPH assay. Although this method provides an initial indication of the antioxidant potential, it reflects only one mechanism of antioxidant action. To achieve a more comprehensive characterization, additional assays such as (2,2′-azino-bis(3-ethylbenzothiazoline-6-sulfonic acid)) assay (ABTS), Ferric Reducing Antioxidant Power (FRAP), and Oxygen Radical Absorbance Capacity (ORAC) should be employed in future studies. Moreover, green propolis was selected as the antioxidant reference in accordance with Veiga et al. [69]. Although vitamin C is more commonly used as the standard in DPPH assays, green propolis offers a phytochemically relevant comparator within the context of this research. Nonetheless, this choice may limit the comparability of results with other studies that use vitamin C as a benchmark.

## 5. Conclusions

In conclusion, the antioxidant, cytocompatibility, and genocompatibility behavior of the extracts from *P. paniculata* and *J. regia* are combined with their ability to inhibit the growth of *F. nucleatum* and *P. micra*, highlighting their potential as therapeutic for intestinal dysbiosis and colorectal cancer risk. This could open new perspectives for the fight against. Nevertheless, further studies are needed to validate their clinical applicability.

## Figures and Tables

**Table 1:** Physicochemical analysis.

Extract	Soluble Solids (g/100 mL)	Total Phenols (mg/100 mL)	Total Flavonoids (mg/100 mL)
*Juglans regia*	6.92 ± 0.27	11.53 ± 0.56	0.04 ± 0.06
*Pfaffia paniculata*	1.93 ± 0.08	7.10 ± 0.95	1.65 ± 0.14

**Table 2:** Antioxidant activity of *J. regia* and *P. paniculata* extracts.

Extract	EC_50_ for *F. nucleatum*	EC_50_ for *P. micra*
*Juglans regia*	37.26 μg/mL	37.26 μg/mL
*Pfaffia paniculata*	1367.57 μg/mL	1367.57 μg/mL

**Table 3:** MIC and MBC analysis of *J. regia* and *P. paniculata* extracts.

Extract/Bacteria	*F. nucleatum*		*P. micra*	
	MIC	MBC	MIC	MBC
*Juglans regia*	nd	1.73 mg/mL	nd	1.73 mg/mL
*Pfaffia paniculata*	nd	0.49 mg/mL	nd	0.49 mg/mL

nd: Not determined.

## Data Availability

The raw data supporting the conclusions of this article will be made available by the authors upon request.

## References

[1] Stokowa-Sołtys K., Kierpiec K., Szczerba K., Wieczorek R. (2023). Can bacteria *F. nucleatum* be actively involved in colon cancer progression via a radical mediated mechanism?. J. Inorg. Biochem..

[2] Bray F., Ferlay J., Soerjomataram I., Siegel R.L., Torre L.A., Jemal A. (2018). Global cancer statistics 2018: GLOBOCAN estimates of incidence and mortality worldwide for 36 cancers in 185 countries. CA Cancer J. Clin..

[3] Pignatelli P., Nuccio F., Piattelli A., Curia M.C. (2023). The Role of *Fusobacterium nucleatum* in Oral and Colorectal Carcinogenesis. Microorganisms.

[4] Petrochilos D., Shojaie A., Gennari J., Abernethy N. (2013). Using random walks to identify cancer-associated modules in expression data. BioData Min..

[5] Martinon P., Fraticelli L., Giboreau A., Dussart C., Bourgeois D., Carrouel F. (2021). Nutrition as a Key Modifiable Factor for Periodontitis and Main Chronic Diseases. J. Clin. Med..

[6] Li R., Shen J., Xu Y. (2022). *Fusobacterium nucleatum* and Colorectal Cancer. Infect. Drug Resist..

[7] Kodio A., Menu E., Ranque S. (2020). Eukaryotic and Prokaryotic Microbiota Interactions. Microorganisms.

[8] Zhao L., Zhang X., Zhou Y., Fu K., Lau H.C.-H., Chun T.W.-Y., Cheung A.H.-K., Coker O.O., Wei H., Wu W.K.-K. (2022). *Parvimonas micra* promotes colorectal tumorigenesis and is associated with prognosis of colorectal cancer patients. Oncogene.

[9] Wong C.C., Yu J. (2023). Gut microbiota in colorectal cancer development and therapy. Nat. Rev. Clin. Oncol..

[10] Cheng Y., Ling Z., Li L. (2020). The Intestinal Microbiota and Colorectal Cancer. Front. Immunol..

[11] Li R., Zhou R., Wang H., Li W., Pan M., Yao X., Zhan W., Yang S., Xu L., Ding Y. (2019). Gut microbiota-stimulated cathepsin K secretion mediates TLR4-dependent M2 macrophage polarization and promotes tumor metastasis in colorectal cancer. Cell Death Differ..

[12] Wang N., Fang J.Y. (2023). *Fusobacterium nucleatum*, a key pathogenic factor and microbial biomarker for colorectal cancer. Trends Microbiol..

[13] Bergsten E., Mestivier D., Donnadieu F., Pedron T., Barau C., Meda L.T., Mettouchi A., Lemichez E., Gorgette O., Chamaillard M. (2023). *Parvimonas micra*, an oral pathobiont associated with colorectal cancer, epigenetically reprograms human colonocytes. Gut Microbes.

[14] Miyakawa Y., Otsuka M., Shibata C., Seimiya T., Yamamoto K., Ishibashi R., Kishikawa T., Tanaka E., Isagawa T., Takeda N. (2024). Gut Bacteria-derived Membrane Vesicles Induce Colonic Dysplasia by Inducing DNA Damage in Colon Epithelial Cells. Cell. Mol. Gastroenterol. Hepatol..

[15] Lam G.A., Albarrak H., McColl C.J., Pizarro A., Sanaka H., Gomez-Nguyen A., Cominelli F., Paes Batista da Silva A. (2023). The Oral-Gut Axis: Periodontal Diseases and Gastrointestinal Disorders. Inflamm. Bowel Dis..

[16] Abed J., Maalouf N., Manson A.L., Earl A.M., Parhi L., Emgård J.E.M., Klutstein M., Tayeb S., Almogy G., Atlan K.A. (2020). Colon Cancer-Associated *Fusobacterium nucleatum* May Originate from the Oral Cavity and Reach Colon Tumors via the Circulatory System. Front. Cell. Infect. Microbiol..

[17] Sun J., Tang Q., Yu S., Xie M., Xie Y., Chen G., Chen L. (2020). Role of the oral microbiota in cancer evolution and progression. Cancer Med..

[18] Baima G., Ferrocino I., Del Lupo V., Colonna E., Thumbigere-Math V., Caviglia G.P., Franciosa I., Mariani G.M., Romandini M., Ribaldone D.G. (2024). Effect of Periodontitis and Periodontal Therapy on Oral and Gut Microbiota. J. Dent. Res..

[19] Löwenmark T., Löfgren-Burström A., Zingmark C., Ljuslinder I., Dahlberg M., Edin S., Palmqvist R. (2022). Tumour colonisation of *Parvimonas micra* is associated with decreased survival in colorectal cancer patients. Cancers.

[20] Chen Y., Yang Y., Gu J. (2020). Clinical implications of the associations between intestinal microbiome and colorectal cancer progression. Cancer Manag. Res..

[21] Conde-Pérez K., Aja-Macaya P., Buetas E., Trigo-Tasende N., Nasser-Ali M., Rumbo-Feal S., Nión P., Arribas E.M., Estévez L.S., Otero-Alén B. (2024). The multispecies microbial cluster of *Fusobacterium*, *Parvimonas*, *Bacteroides* and *Faecalibacterium* as a precision biomarker for colorectal cancer diagnosis. Mol. Oncol..

[22] Xu J., Yang M., Wang D., Zhang S., Yan S., Zhu Y., Chen W. (2020). Alteration of the abundance of *Parvimonas micra* in the gut along the adenoma-carcinoma sequence. Oncol. Lett..

[23] Boehnke K.F., Valdivieso M., Bussalleu A., Sexton R., Thompson K.C., Osorio S., Novoa Reyes I., Crowley J.J., Baker L.H., Xi C. (2017). Antibiotic resistance among Helicobacter pylori clinical isolates in Lima, Peru. Infect. Drug Resist..

[24] Sandu-Bălan Tăbăcariu A., Ifrim I.L., Patriciu O.I., Ștefănescu I.A., Fînaru A.L. (2024). Walnut By-Products and Elderberry Extracts-Sustainable Alternatives for Human and Plant Health. Molecules.

[25] Mateș L., Rusu M.E., Popa D.S. (2023). Phytochemicals and Biological Activities of Walnut Septum: A Systematic Review. Antioxidants.

[26] Acquaviva R., D’Angeli F., Malfa G.A., Ronsisvalle S., Garozzo A., Stivala A., Ragusa S., Nicolosi D., Salmeri M., Genovese C. (2021). Antibacterial and anti-biofilm activities of walnut pellicle extract (*Juglans regia* L.) against coagulase-negative staphylococci. Nat. Prod. Res..

[27] Farooqui A., Khan A., Borghetto I., Kazmi S.U., Rubino S., Paglietti B. (2015). Synergistic Antimicrobial Activity of *Camellia sinensis* and *Juglans regia* against Multidrug-Resistant Bacteria. PLoS ONE.

[28] Li C., Zhang Z., Zhang S., Yan W., Si C., Lee M.H., Li Z. (2019). Inhibitory Effects of the Extracts of *Juglans sigillata* Green Husks on the Proliferation, Migration and Survival of KYSE150 and EC9706 Human Esophageal Cancer Cell Lines. Nutr. Cancer.

[29] Oniyangi O., Cohall D.H. (2020). Phytomedicines (medicines derived from plants) for sickle cell disease. Cochrane Database Syst. Rev..

[30] Costa C.A.R.A., Quaglio A.E.V., Di Stasi L.C. (2018). *Pfaffia paniculata* (*Brazilian ginseng*) extract modulates Mapk and mucin pathways in intestinal inflammation. J. Ethnopharmacol..

[31] Costa C.A., Tanimoto A., Quaglio A.E., Almeida L.D., Severi J.A., Di Stasi L.C. (2015). Anti-inflammatory effects of *Brazilian ginseng* (*Pfaffia paniculata*) on TNBS-induced intestinal inflammation: Experimental evidence. Int. Immunopharmacol..

[32] da Silva T.C., Cogliati B., Latorre A.O., Akisue G., Nagamine M.K., Haraguchi M., Hansen D., Sanches D.S., Dagli M.L.Z. (2015). Pfaffosidic Fraction from Hebanthe paniculata Induces Cell Cycle Arrest and Caspase-3-Induced Apoptosis in HepG2 Cells. Evid.-Based Complement. Altern. Med..

[33] Nagamine M.K., da Silva T.C., Matsuzaki P., Pinello K.C., Cogliati B., Pizzo C.R., Akisue G., Haraguchi M., Górniak S.L., Sinhorini I.L. (2009). Cytotoxic effects of butanolic extract from *Pfaffia paniculata* (*Brazilian ginseng*) on cultured human breast cancer cell line MCF-7. Exp. Toxicol. Pathol..

[34] Bourais I., Elmarrkechy S., Taha D., Badaoui B., Mourabit Y., Salhi N., Alshahrani M.M., Al Awadh A.A., Bouyahya A., Goh K.W. (2022). Comparative investigation of chemical constituents of kernels, leaves, husk, and bark of *Juglans regia* L., using HPLC-DAD-ESI-MS/MS analysis and evaluation of their antioxidant, antidiabetic, and anti-inflammatory activities. Molecules.

[35] Li J., Jadhav A.N., Khan I.A. (2010). Triterpenoids from *Brazilian ginseng*, *Pfaffia paniculata*. Planta Medica.

[36] Rodrigues M.V.N., Souza K.P., Rehder V.L.G., Vilela G.F., Montanari Junior I., Figueira G.M., Rath S. (2013). Development of na analytical method for the quantification of pfaffic acid in Brazilian ginseng (*Hebanthe eriantha*). J. Pharm. Biomed. Anal..

[37] Alcázar Magaña A., Kamimura N., Soumyanath A., Stevens J.F., Maier C.S. (2021). Caffeoylquinic acids: Chemistry, biosynthesis, occurrence, analytical challenges, and bioactivity. Plant J..

[38] Diemer E., Chadni M., Grimi N., Ioannou I. (2022). Optimization of the Accelerated Solvent Extraction of Caffeoylquinic Acids from Forced Chicory Roots and Antioxidant Activity of the Resulting Extracts. Foods.

[39] Makori S.I., Mu T.H., Sun H.N. (2021). Physicochemical properties, antioxidant activities, and binding behavior of 3,5-di-O-caffeoylquinic acid with beta-lactoglobulin colloidal particles. Food Chem..

[40] Rojas-González A., Figueroa-Hernández C.Y., González-Rios O., Suárez-Quiroz M.L., González-Amaro R.M., Hernández-Estrada Z.J., Rayas-Duarte P. (2022). Coffee Chlorogenic Acids Incorporation for Bioactivity Enhancement of Foods: A Review. Molecules.

[41] Naveed M., Hejazi V., Abbas M., Kamboh A.A., Khan G.J., Shumzaid M., Ahmad F., Babazadeh D., FangFang X., Mo-darre-si-Ghazani F. (2018). Chlorogenic acid (CGA): A pharmacological review and call for further re-search. Biomed Pharmacother..

[42] Fiamegos Y.C., Kastritis P.L., Exarchou V., Han H., Bonvin A.M., Vervoort J., Lewis K., Hamblin M.R., Tegos G.P. (2011). Antimicrobial and efflux pump inhibitory activity of caffeoylquinic acids from *Artemisia absinthium* against gram-positive pathogenic bacteria. PLoS ONE.

[43] Khan R., Naseem I. (2023). Antiglycation and antioxidant potential of coumaric acid isomers: A comparative in-vitro study. J. Biomol. Struct. Dyn..

[44] Morales J., Mendoza L., Cotoras M. (2017). Alteration of oxidative phosphorylation as a possible mechanism of the antifungal action of p-coumaric acid against Botrytis cinerea. J. Appl. Microbiol..

[45] Lopes S.P., Castillo Y.P., Monteiro M.L., Menezes R.R.P.P.B., Almeida R.N., Martins A.M.C., Sousa D.P. (2019). Trypanocidal Mechanism of Action and in silico Studies of p-Coumaric Acid Derivatives. Int. J. Mol. Sci..

[46] Costa P., Boeing T., Somensi L.B., Cury B.J., Espíndola V.L., França T.C.S., de Almeida M.O., Arruda C., Bastos J.K., da Silva L.M. (2019). Hydroalcoholic extract from *Baccharis dracunculifolia* recovers the gastric ulcerated tissue, and p-coumaric acid is a pivotal bioactive compound to this action. BioFactors.

[47] Ojha D., Patil K.N. (2019). p-Coumaric acid inhibits the Listeria monocytogenes RecA protein functions and SOS response: An antimi-crobial target. Biochem. Biophys. Res. Commun..

[48] Mozar A., Charlot K., Sandor B., Rabaï M., Lemonne N., Billaud M., Hardy-Dessources M.-D., Beltan E., Pandey R.C., Connes P. (2015). *Pfaffia paniculata* extract improves red blood cell de-formability in sickle cell patients. Clin. Hemorheol. Microcirc..

[49] Ochensberger S., Alperth F., Mitić B., Kunert O., Mayer S., Mourão M.F., Turek I., Luca S.V., Skalicka-Woźniak K., Maleš Ž. (2019). Phenolic compounds of Iris adriatica and their antimyco-bacterial effects. Acta Pharm..

[50] Lima A.M.A., Teixeira T.R., Silva B.F.S., Raoni P., Silva I.E.P., Santos E.G., Fernandes M.C., Gonçalves V., Bressan G., Mendes T. (2019). Síntese e avaliação das atividades fotoprotetora, citotóxica e antiviral contra o zika vírus de derivados triazólicos da benzofenona. Quim. Nova.

[51] Bai M., Gao C.H., Liu K., Zhao L.Y., Tang Z.Z., Liu Y.H. (2021). Two new benzophenones isolated from a mangrove-derived fungus *Penicillium* sp.. J. Antibiot..

[52] Ji Y.B., Chen W.J., Shan T.Z., Sun B.Y., Yan P.C., Jiang W. (2020). Antibacterial Diphenyl Ether, Benzophenone and Xanthone Derivatives from *Aspergillus flavipes*. Chem. Biodivers..

[53] Morrissey J.P., Osbourn A.E. (1999). Fungal Resistance to Plant Antibiotics as a Mechanism of Pathogenesis. Microbiol. Mol. Biol. Rev..

[54] Chakraborty A.K., Saha S., Poria K., Samanta T., Gautam S., Mukhopadhyay J. (2022). A saponin-polybromophenol antibiotic (CU1) from Cassia fistula Bark Against Multi-Drug Resistant Bacteria Targeting RNA polymerase. Curr. Res. Pharmacol. Drug Discov..

[55] Sun X., Yang X., Xue P., Zhang Z., Ren G. (2019). Improved antibacterial effects of alkali-transformed saponin from quinoa husks against halitosis-related bacteria. BMC Complement. Altern. Med..

[56] Silva L.N., Trentin Dda S., Zimmer K.R., Treter J., Brandelli C.L., Frasson A.P., Tasca T., da Silva A.G., da Silva M.V., Macedo A.J. (2015). Anti-infective effects of Brazilian Caatinga plants against pathogenic bacterial biofilm formation. Pharm. Biol..

[57] Coêlho M.L., Ferreira J.H., de Siqueira Júnior J.P., Kaatz G.W., Barreto H.M., de Carvalho Melo Cavalcante A.A. (2016). Inhibition of the NorA multi-drug transporter by oxygenated monoterpenes. Microb. Pathog..

[58] Bezerra L.F.G., Silva A.P.S.D., Cunha R.X.D., Oliveira J.R.S., Barros M.D., Silva V.M.M.A.D., Lima V.L.M. (2023). Antioxidant, anti-inflammatory and analgesic activity of *Mimosa acutistipula* (Mart.) Benth. J. Ethnopharmacol..

[59] Żurek N., Pawłowska A., Pycia K., Grabek-Lejko D., Kapusta I.T. (2022). Phenolic Profile and Antioxidant, Antibacterial, and Antiproliferative Activity of *Juglans regia* L. Male Flowers. Molecules.

[60] Eberlin S., Del Carmen Velazquez Pereda M., de Campos Dieamant G., Nogueira C., Werka R.M., de Souza Queiroz M.L. (2009). Effects of a Brazilian herbal compound as a cosmetic eyecare for periorbital hyperchromia (“dark circles”). J. Cosmet. Dermatol..

[61] Huang X., Li Y., Wang T., Liu H., Shi J., Zhang X. (2020). Evaluation of the Oxidative Stress Status in Zebrafish (*Danio rerio*) Liver Induced by Three Typical Organic UV Filters (BP-4, PABA and PBSA). Int. J. Environ. Res. Public Health.

[62] Mohammed A.E., Aabed K., Benabdelkamel H., Shami A., Alotaibi M.O., Alanazi M., Alfadda A.A., Rahman I. (2023). Proteomic Pro-fil-ing Reveals Cytotoxic Mechanisms of Action and Adaptive Mechanisms of Resistance in *Porphyromonas gingivalis*: Treat-ment with *Juglans regia* and *Melaleuca alternifolia*. ACS Omega.

[63] Pham Q.D., Topgaard D., Sparr E. (2015). Cyclic and Linear Monoterpenes in Phospholipid Membranes: Phase Behavior, Bilayer Structure, and Molecular Dynamics. Langmuir.

[64] de Groot C., Müller-Goymann C.C. (2016). Saponin Interactions with Model Membrane Systems—Langmuir Monolayer Studies, Hemolysis and Formation of ISCOMs. Planta Medica.

[65] Sakanaka A., Kuboniwa M., Shimma S., Alghamdi S.A., Mayumi S., Lamont R.J., Fukusaki E., Amano A. (2022). *Fusobacterium nucleatum* Metabolically Integrates Commensals and Pathogens in Oral Biofilms. mSystems.

[66] Adnan M., Siddiqui A.J., Ashraf S.A., Ashraf M.S., Alomrani S.O., Alreshidi M., Tepe B., Sachidanandan M., Danciu C., Patel M. (2023). Sapo-nin-Derived Silver Nanoparticles from *Phoenix dactylifera* (Ajwa Dates) Exhibit Broad-Spectrum Bioactivities Combating Bacterial Infections. Antibiotics.

[67] Rahamouz-Haghighi S., Sharafi A. (2023). Antiproliferative assay of suma or *Brazilian ginseng* (*Hebanthe eriantha*) methanolic extract on HCT116 and 4T1 cancer cell lines, in vitro toxicity on Artemia salinalarvae, and antibacterial activity. Nat. Prod. Res..

[68] Lee J.B., Kim K.A., Cho H.Y., Kim D., Kim W.K., Yong D., Lee H., Yoon S.S., Han D.H., Han Y.D. (2021). Association between *Fusobacterium nucleatum* and patient prognosis in metastatic colon cancer. Sci. Rep..

[69] Veiga R.S., De Mendonça S., Mendes P., Paulino N., Mimica M., Netto A.L., Lira I., López B.-C., Negrão V., Marcucci M. (2017). Artepillin C and phenolic compounds responsible for antimicrobial and antioxidant activity of green propolis and *Baccharis dracunculifolia* DC. J. Appl. Microbiol..

